# Homeostatic modulation on unconscious hedonic responses to food

**DOI:** 10.1186/s13104-017-2835-y

**Published:** 2017-10-26

**Authors:** Wataru Sato, Reiko Sawada, Yasutaka Kubota, Motomi Toichi, Tohru Fushiki

**Affiliations:** 1 Department of Neurodevelopmental Psychiatry, Habilitation and Rehabilitation, Graduate School of Medicine, Kyoto University, 53 Shogoin-Kawaharacho, Sakyo, Kyoto 606-8507 Japan; 2 Health and Medical Services Center, Shiga University, 1-1-1 Baba, Hikone, Shiga 522-8522 Japan; 3 Faculty of Human Health Science, Graduate School of Medicine, Kyoto University, 53 Shogoin-Kawaharacho, Sakyo-ku, Kyoto, 606-8507 Japan; 4 The Organization for Promoting Neurodevelopmental Disorder Research, 40 Shogoin-Sannocho, Sakyo, Kyoto 606-8392 Japan; 5 Faculty of Agriculture, Ryukoku University, 1-5 Seta Oe-Cho Koya, Ohtsu, Shiga 520-2194 Japan

**Keywords:** Dutch eating behavior questionnaire (DEBQ), Food, Hungry–full homeostatic states, Subliminal affective priming, Unconscious hedonic responses

## Abstract

**Objective:**

Hedonic/affective responses to food play a critical role in eating behavior. Previous behavioral studies have shown that hedonic responses to food are elicited consciously and unconsciously. Although the studies also showed that hunger and satiation have a modulatory effect on conscious hedonic responses to food, the effect of these homeostatic states on unconscious hedonic responses to food remains unknown.

**Results:**

We investigated unconscious hedonic responses to food in hungry and satiated participants using the subliminal affective priming paradigm. Food images or corresponding mosaic images were presented in the left or right peripheral visual field during 33 ms. Then photographs of target faces with emotionally neutral expressions were presented, and the participants evaluated their preference for the faces. Additionally, daily eating behaviors were assessed using questionnaires. Preference for the target faces was increased by food images relative to the mosaics in the hungry, but not the satiated, state. The difference in preference ratings between the food and mosaic conditions was positively correlated with the tendency for external eating in the hungry, but not the satiated, group. Our findings suggest that homeostatic states modulate unconscious hedonic responses to food and that this phenomenon is related to daily eating behaviors.

**Electronic supplementary material:**

The online version of this article (doi:10.1186/s13104-017-2835-y) contains supplementary material, which is available to authorized users.

## Introduction

Hedonic or affective responses, such as liking, to food stimuli have significant effects on human life, both advantageously (e.g., facilitating survival) and disadvantageously (e.g., triggering overeating and lifestyle disease). Previous behavioral studies have shown that seeing and consuming food elicit hedonic responses, which in turn induce food intake [1–8].

A recent study has demonstrated that hedonic responses to food were elicited rapidly even without conscious awareness of the food [9] using a subliminal affective priming paradigm [10, 11]. The investigators found that the subliminal presentation of food stimuli heightened the preference for subsequent face targets more than the subliminal presentation of mosaics. These findings suggest that the unconscious hedonic responses elicited by food spill over into the hedonic evaluation of unrelated targets. Such behavioral evidence is consistent with findings from neuroimaging studies showing that brain regions that are activated in response to the sight of food, such as the amygdala and nucleus accumbens [e.g., [12]; for a review, see [13] ], are also activated in response to subliminally presented non-food affective stimuli, such as emotional facial expressions [14]. Taken together, previous behavioral and neuroimaging data suggest that hedonic responses to food are elicited both consciously and unconsciously.

However, whether the unconscious hedonic responses to food might be modulated by the homeostatic states of hunger and satiation remains unknown. This issue is important because hedonic responses to external food stimuli and homeostatic control of internal states jointly control eating behaviors [15, 16]. A previous study has tested unconscious hedonic responses to food only in hungry participants [9]; thus, the unconscious hedonic response to food in the satiated state remains unknown. In contrast, several previous studies fund that conscious hedonic responses to food are modulated by hunger and satiation [2–4, 17–20]; specifically, the pleasant feelings toward supraliminal food stimuli are reduced in satiated compared with hungry participants [2–4, 17–20]. Moreover, the rapid, automatic processes related to supraliminal food stimuli, such as attention orienting to food, are attenuated in the satiated relative to the hungry state [18, 21–23]. Furthermore, neuroimaging studies showed reduced amygdala activity during the observation of supraliminal food images in the satiated compared with the hungry state [24, 25]. Based on these data, we hypothesized that unconscious hedonic responses to food may be modulated by the homeostatic state such that the responses are attenuated in the satiated relative to the hungry state.

Furthermore, it remains unclear whether the relationship between unconscious hedonic responses to food and daily eating behaviors could be modulated by homeostatic states. A previous investigation of unconscious hedonic responses to food [9] has assessed daily eating behaviors using the Dutch eating behavior questionnaire (DEBQ) [26]. The DEBQ measures the tendency for external eating, restrained eating, and emotional eating toward overeating [26]. The investigators found that the unconscious preference for food was correlated positively with external eating, defined as a tendency to eat in response to external cues, such as the sight of food. However, this study did not include fed participants. A previous neuroimaging study found that external eating modulated the functional connectivity between the amygdala and nucleus accumbens during the viewing of food images [27]. Because amygdala activity is altered according to homeostatic state [24, 25], we hypothesized that the relationship between unconscious hedonic responses to food and external eating tendencies may be modulated by homeostatic state.

**Fig. 1:**
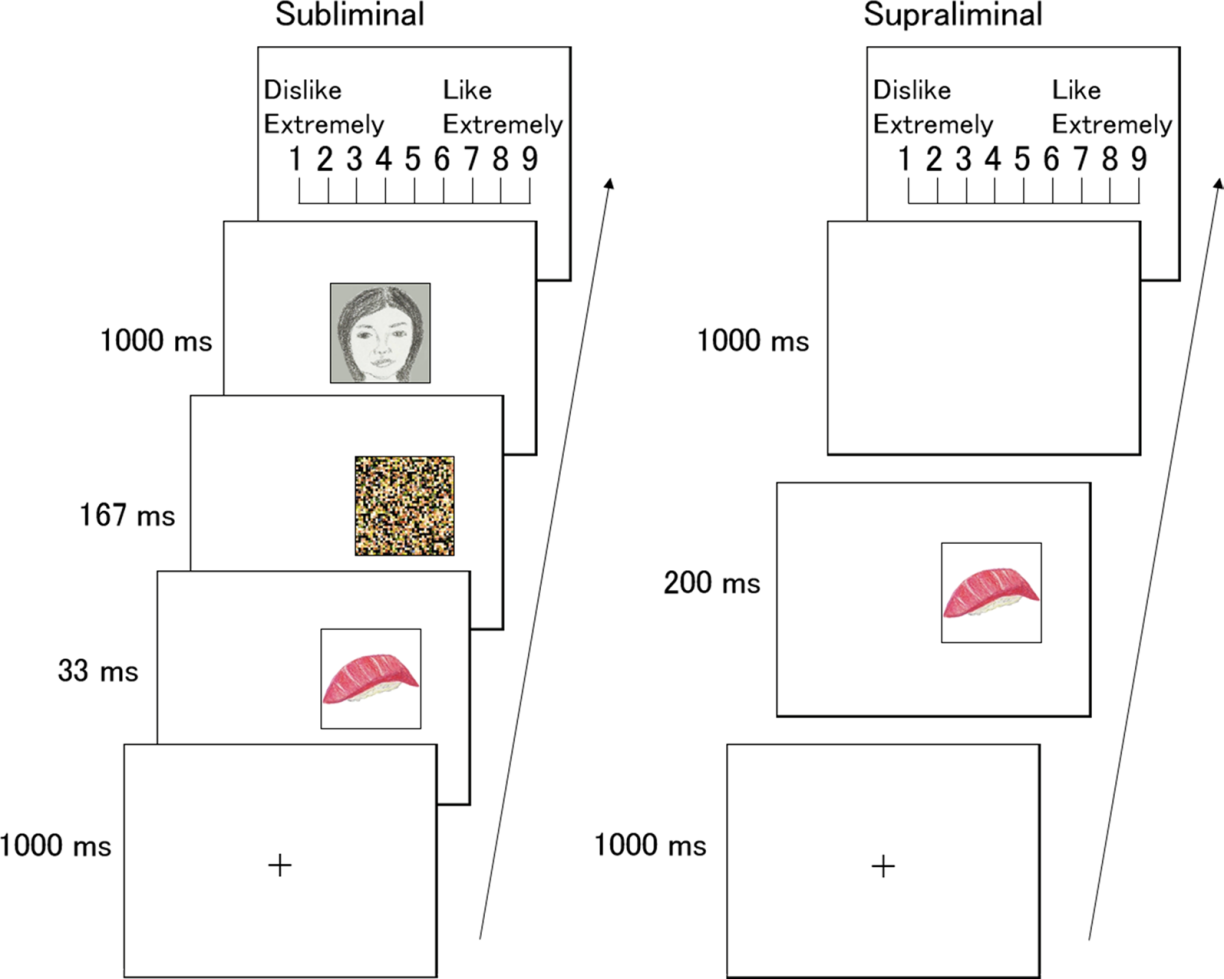
Schematic of the protocols under the subliminal and supraliminal presentation conditions. Photographic stimuli were used in the experiments

To test theses hypotheses, we investigated the unconscious and conscious hedonic responses to food and non-food images in hungry and satiated participants. We assessed the unconscious hedonic responses using the subliminal affective priming paradigm [28] (Fig. 1). We presented food images and corresponding mosaics during 33 ms in the inattentional peripheral visual field, followed by a mask. Target faces with neutral expressions were subsequently presented, and participants evaluated their preference for the faces. To investigate conscious hedonic responses, we supraliminally presented food and mosaic images and participants rated their preference for the images. Participants were randomly assigned to the hungry- or satiated-state condition and we tested the modulatory effect of the homeostatic state on unconscious and conscious hedonic responses to food. Moreover, we used the DEBQ and evaluated the modulatory influence of homeostatic state on the relationships between the unconscious/conscious hedonic responses and daily eating behaviors.

## Main text

Methods and Supplementary findings are presented in Additional files 1 and 2, respectively.

## Findings

### Preference evaluation

**Fig. 2:**
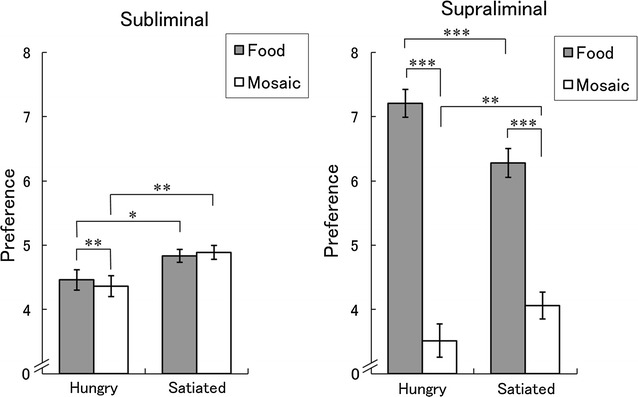
Mean (± standard error) preference ratings under the subliminal (left) and supraliminal (right) presentation conditions. Asterisks indicate significant simple main effects (*p < 0.05; **p < 0.005; ***p < 0.001)

Under the subliminal condition, the two-way analysis of variance (ANOVA) for preference ratings for target faces (Fig. 2), with homeostatic state (hungry/satiated) and stimulus type (food/mosaic) as factors, revealed a significant main effect of homeostatic state (*F*(1,67) = 6.74, *p* < 0.05, *η*_*p*_^2^ = 0.09), indicating that the preference ratings for faces under the satiated condition were higher than those under the hungry condition. Furthermore, the interaction between homeostatic state and stimulus type was significant (*F*(1,67) = 7.19, *p* < 0.005, *η*_*p*_^2^ = 0.10), indicating that the subliminal hedonic effects of food differed between homeostatic states. The main effect of stimulus type was not significant (*F*(1,67) = 1.84, *p* > 0.1).

Follow-up analyses of the interaction revealed a significant simple main effect of stimulus type under the hungry (*F*(1,67) = 8.15, *p* < 0.005), but not under the satiated (*F*(1,67) = 0.87, *p* > 0.1), condition, indicating that the preference ratings for faces primed by food images were higher than those primed by mosaics under the hungry, but not the satiated, condition. The simple main effect of homeostatic state was significant for the food and mosaic stimuli (*F*(1,134) = 4.77, *p* < 0.05; *F*(1,134) = 8.73, *p* < 0.005, respectively) confirming the main effect of homeostatic state.

Under the supraliminal condition, the two-way ANOVA for preference ratings for food and mosaic stimuli (Fig. 2) revealed a significant main effect of stimulus type (*F*(1,67) = 267.73, *p* < 0.001, *η*_*p*_^2^ = 0.80), indicating that the preference ratings for food were higher than those for the mosaics. More importantly, the interaction between homeostatic state and stimulus type was significant (*F*(1,67) = 14.25, *p* < 0.001, *η*_*p*_^2^ = 0.18), indicating that the preferential ratings for food versus mosaics differed between homeostatic states. The main effect of homeostatic state was not significant (*F*(1,67) = 1.84, *p* > 0.1).

Follow-up analyses of the interaction revealed a significant simple main effect of stimulus type under the hungry and satiated conditions (*F*(1,67) = 202.75, *p* < 0.001; *F*(1,67) = 79.227, *p* < 0.001, respectively), indicating that the preference ratings for food were higher than those for mosaics in both homeostatic states. The simple main effect of homeostatic state was also significant for the food and mosaic stimuli (*F*(1,134) = 8.75, *p* < 0.001; *F*(1,134) = 4.40, *p* < 0.005, respectively), indicating that the preference for food stimuli was decreased in the satiated state and the preference for mosaic stimuli was decreased in the hungry state.

### Relationship between food preference and daily eating behaviors

**Fig. 3:**
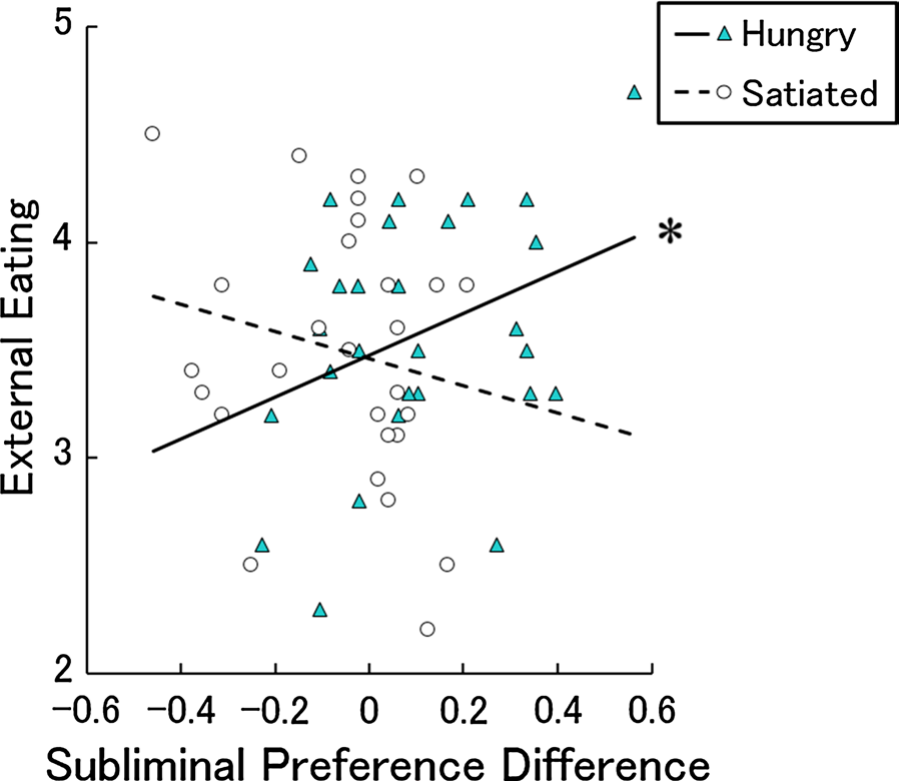
A scatterplot with a regression line showing food preference scores under the subliminal condition as a function of external eating tendencies. An asterisk indicates a significant correlation (*p < 0.05)

We calculated the correlation coefficients between the food preference (differences in preference ratings between the food and mosaic conditions) and the DEBQ scores under the subliminal and supraliminal conditions (Additional file 3: Table S1). Consistent with our hypothesis, the correlation coefficients between the food preference under the subliminal condition and external eating scores differed for the different homeostatic states (Fig. 3). They were significant in the hungry state (*r* = 0.34, *p* < 0.05), but not in the satiated state (*r* = − 0.19, *p* > 0.1). To statistically test this difference, we performed a multiple regression analysis with the external eating score, homeostatic state, and the interaction between them as independent variables and the food preference under the subliminal condition as the dependent variable and confirmed the significant interaction (*β* = 0.26, *t*(52) = 2.08, *p* < 0.05). We conducted a series of explorative regression analyses and found no other significant interaction between homeostatic state and DEBQ score (*t*(52) < 1.60, *p* > 0.1).

## Discussion

Our findings revealed a modulatory effect of homeostatic state (hunger versus satiation) on preference ratings for food and mosaic images under the supraliminal condition, such that participants in the satiated group showed a decreased preference for food and increased preference for mosaics relative to those in the hungry group. This pattern corroborates previous findings that the satiated state is associated with a decreased preference for food and increased preference for non-food items [3, 4, 19].

More importantly, our findings under the subliminal condition revealed that compared with the hungry state, satiation attenuated the unconscious hedonic responses to food (interaction between homeostatic state and stimulus type) and heightened the overall preference for faces (main effect of homeostatic state). Our findings are consistent with those of previous studies using supraliminal stimuli [2–4, 17–20]. Furthermore, our findings do not contradict those of neuroimaging studies showing that less amygdala activity was elicited by supraliminal presentation of food stimuli in the satiated state than in the hungry state [24, 25]. Moreover, a recent neuroimaging study [29] found that amygdala activity elicited by the supraliminal presentation of food stimuli was modulated by hypothalamic input in hungry, but not satiated, participants. The hypothalamus is known to be involved in homeostatic regulation via communication with the somatic system [30]. Our study extends previous findings related to conscious hedonic responses by providing the first evidence that unconscious hedonic responses to food are modulated by homeostatic states.

Furthermore, our correlational analyses revealed that the positive association between the food preference and external eating under the subliminal condition was weaker in the satiated compared with the hungry participants. Our finding is consistent with those in previous neuroimaging studies showing that (1) amygdala activity is related to the unconscious hedonic processing of non-food stimuli [14], (2) amygdala functional connectivity is modulated by external eating tendencies [27], and (3) food-related amygdala activity is altered in response to homeostatic state [24, 25]. Because the external eating score is an indicator of the tendency for eating to be elicited by external stimuli [26], our behavioral findings indicate that externally driven daily eating behaviors are associated with unconscious hedonic responses specifically when individuals are hungry.

Our study has several practical implications. First, our findings suggest that hunger and satiation may influence unconscious hedonic responses to food and daily eating behaviors related to overeating. Because healthy eating behaviors, such as consuming a low-calorie diet, require suppressing unconscious hedonic responses and externally-driven overeating behaviors, our findings may provide behavioral strategies for dieting. For example, we recommend that people eat before visiting food-abundant environments, such as supermarkets and convenience stores, to reduce the unconscious hedonic processing of food and subsequent overeating.

Second, because images of faces were the target stimuli under the subliminal condition, our results imply that being satiated unconsciously enhances the general preference for people. Thus, the consumption of food during meetings, on romantic dates, or in various other situations may unconsciously heighten emotionally positive interactions.

## Limitations

Our study has several limitations. First, we used a between-subjects experimental design to examine the effect of homeostatic state. Although this design allowed us to investigate the participants’ naïve responses (without contextual referencing) under the subliminal condition [31], it did not allow us to assess whether changes in homeostatic states could modulate unconscious hedonic responses, as well as the association between unconscious hedonic responses and external eating tendencies, in individual participants as would a within-subjects design. Further studies using a within-subjects design are warranted to complement and extend our findings.

Second, we only assessed daily eating behaviors using the DEBQ and did not measure actual food intake behaviors. The relationship between the DEBQ scores and actual food intake behaviors are controversial; some investigators have reported convergence between these measures [e.g., [32]] while others have not [e.g., [33]]. The relationship between unconscious homeostatic–hedonic interaction and actual food intake behaviors is an interesting topic for future research.

## Additional files

**Additional file 1.** Methods. Description of methods.

**Additional file 2.** Supplementary findings. Findings from additional analysis.

**Additional file 3: Table S1.** Mean (± standard error) scores and the correlation coefficients.

**Additional file 4.** Dataset. Raw data.

## Abbreviations

ANOVA: analysis of variance
DEBQ: Dutch eating behavior questionnaire
