# Supplementary material for: Homeostatic modulation on unconscious hedonic responses to food
Source: BMC Res Notes. 2017 Oct 26;10:511. doi: 10.1186/s13104-017-2835-y (PMC5658914; doi:10.1186/s13104-017-2835-y)
Supplement: Supplementary file 1 — Additional file 1. Methods. Description of methods. [file 13104_2017_2835_MOESM1_ESM.pdf]

Additional File 1: Methods

Participants

The study included 56 healthy Japanese volunteers, who were randomly assigned to the hungry-state (13 females and 15 males; $M \pm SD$ age, 22.9 ± 4.4 years; $M \pm SD$ body mass index (BMI), 21.0 ± 2.3 kg/m^2) or the satiated-state (13 females and 15 males; $M \pm SD$ age, 23.4 ± 4.7 years; $M \pm SD$ BMI, 20.8 ± 1.5 kg/m^2) condition. Hunger level ratings were significantly higher in the hungry ($M \pm SE$, 4.2 ± 0.1) than in the satiated ($M \pm SE$, 1.5 ± 0.1) participants ($t(67) = 5.92$, $p < 0.001$). The hungry and satiated participants were matched for sex (χ^2 test, $p > 0.1$), age (t-test, $p > 0.1$), BMI (t-test, $p > 0.1$), and Dutch Eating Behavior Questionnaire (DEBQ) scores (t-test, $p > 0.1$). The hungry group was required to fast for at least 3 h before the experiment and satiated group was tested within 30 min of the last meal. Some additional participants who reported seeing food images under the subliminal presentation condition ($n = 7$) or who rated their hunger level as neutral ($n = 6$) were excluded from the analysis. All participants had normal or corrected-to-normal visual acuity. Written informed consent was obtained from all participants after the experimental procedure was fully explained. This study was approved by the Ethics Committee of the Graduate School of Medicine, Kyoto University, and was conducted in accordance with the approved guidelines. The data of some participants in the hungry-state group were included in a previous study [1].

Experimental design

The preference rating experiment used a two-factorial repeated-measures design, with homeostatic state (hungry or satiated) as a between-subjects factor and stimulus

type (food or mosaic) as a within-subjects factor. Implicit and explicit preference ratings were conducted under the subliminal and supraliminal presentation conditions, respectively.

Stimuli

The food stimuli were color photographs of 12 fast food items (e.g., hamburgers) and 12 traditional Japanese diet items (e.g., sushi). The photographs were selected from among images that appeared on websites and were cropped using PhotoShop CS6 (Adobe, San Jose, CA). The size of the stimuli was 7.0° vertically × 7.0° horizontally.

The mosaic stimuli were constructed from the food stimuli using Matlab 6.5 (Mathworks, Natick, MA). First, all of the food stimuli were cut into small squares (40 vertical × 40 horizontal) and reordered using a randomization algorithm. The rearrangement rendered each image unrecognizable as food. A mask stimulus was created from a mosaic pattern composed of fragments of food images not used in the experiment.

Face images were prepared as target stimuli for the implicit preference rating task. Faces were grayscale photographs depicting full-face neutral expressions of 48 (24 female and 24 male) Japanese models. The target stimuli were randomly assigned to conditions and appeared once in each of the first and second blocks. The face stimuli were 7.0° vertically × 7.0° horizontally.

Apparatus

The stimulus presentation was controlled using Presentation 14.9 (Neurobehavioral

Systems, Albany, CA) implemented on a Windows computer (HP Z200 SFF; Hewlett-Packard, Tokyo, Japan). The stimuli were presented on a 19-inch CRT monitor (HM903D-A; Iiyama, Tokyo, Japan) with a refresh rate of 150 Hz and a resolution of 1024 × 768 pixels.

Questionnaires

We used the Japanese version of the DEBQ [2, 3] to measure daily eating behaviors. The questionnaire included 33 items that assessed three tendencies related to overeating: restrained eating (10 items; e.g., "Do you deliberately eat less in order to not become heavier?"), emotional eating (13 items, e.g., "Do you have the desire to eat when you are irritated?"), and external eating (10 items, e.g., "If you see or smell something delicious, do you have a desire to eat it?"). Each item was scored on a scale ranging from 1 (*seldom*) to 5 (*very often*). The internal consistency reliability (Cronbach's $\alpha > 0.83$) and factor validity (the same three factors) of the Japanese versions of the DEBQ were confirmed [3].

Additionally, we assessed the hunger level of participants on a 5-point scale ranging from 1 (*not at all*) to 5 (*very*) and provided an electric calculator for participants to calculate their BMI based on an equation that was provided to them.

Procedure

The experiments were conducted individually for each participant. On arrival, the participant was told that the experiment concerned preference evaluations related to people and food; they were to complete questionnaires, including the one pertaining to hunger level. Then the participant was seated 0.57 m from the monitor. The

subliminal condition preceded the supraliminal condition.

Under each subliminal and supraliminal presentation condition, a total of 96 trials involving preference judgments (48 food and 48 mosaic) were performed. Participants completed these trials in two blocks of 48. Each block contained an equal number of trials with each of the stimulus type/food type/visual field conditions. The order of conditions was randomized within each block. Participants were given a short break after each block and a longer break after completion of the subliminal condition. The participants performed five practice trials to become familiar with the procedure under each presentation condition before testing began.

In each trial under the subliminal presentation condition (Fig. 1), a cross was initially presented for 1,000 ms as a fixation point at the center of the visual field. A prime stimulus was then presented for 33 ms in the left or the right visual field (the inside edge was 3.5° peripheral to the center) immediately followed by the presentation of a mask stimulus in the same location for 167 ms. The exposure duration of the prime and mask stimuli were based on data from previous studies using subliminal presentations [4] and the results of our own preliminary studies. The target face was presented immediately after the mask stimulus in the central visual field for 1,000 ms. Finally, the rating display was presented and remained until the participant entered a response. Participants were instructed to maintain their gaze at the location where the fixation point had appeared throughout the trial. The participants' task was to rate their preference for the target faces using a 9-point scale ranging from "dislike extremely" to "like extremely" [5]. They were asked to respond by pressing keys with their right index finger.

In each trial under the supraliminal presentation condition (Fig. 1), after a cross

appeared for 1,000 ms as a fixation point at the center of the visual field, a target food/mosaic image was presented for 200 ms in the left or the right visual field (the inside edge was 3.5° peripheral to the center). After the presentation of a blank screen for 1,000 ms, the rating display was presented and remained until the participant entered a response. Participants were instructed to maintain their gaze at the location of the fixation point throughout the trial. Their task was to rate their preference for the target food/mosaic images in the same manner as they did for faces under the subliminal presentation condition.

As in previous studies [6, 7], participants performed the forced-choice discrimination task after completion of the preference ratings. A total of 48 trials were conducted using food stimuli. In each trial, the sequence of events was the same as those under the subliminal presentation condition. Then, the two food stimuli, one of which had been presented as the prime, were presented in the upper and lower visual fields, and the participants were asked to select the food image that had been presented. Both stimuli were from the same food subcategory. One-sample *t*-tests performed to detect differences above the level of chance revealed that the percentage of correct discriminations under the hungry ($M \pm SE$, 52.5 ± 2.6%) and satiated ($M \pm SE$, 51.8 ± 3.2%) conditions was not significantly different from chance ($t < 1.20$, $p > 0.1$). Thus, our experimental conditions met the objective criteria set out for the subliminal presentation of stimuli [8].

After completing the forced-choice discrimination task, the participants were asked to complete the DEBQ and BMI questionnaires. In a subsequent interview, they were asked whether they had consciously perceived the primes under the subliminal presentation condition. The participants were then debriefed. After

explaining the purpose of the experiment, we requested the participants' permission to use their data in our analysis, and all of the participants consented to this request.

Data analysis

The statistical analyses were conducted using SPSS 16.0J (SPSS, Tokyo, Japan). The preference rating data were analyzed separately for each presentation condition using a two-way repeated-measures analysis of variance (ANOVA) with homeostatic state (hungry/satiated) and stimulus type (food/mosaic) as factors. Simple-effect analyses were performed as follow-up analyses [9]. Because the sex, age, food category, and visual field factors were not of interest in this study and our preliminary analyses showed that the interactions between homeostatic state and stimulus type were retained when these factors were partialed out, we presented the findings regarding these factors in Supplementary Findings.

To investigate the relationship between hedonic responses to food and eating behaviors, differences between the preference ratings for the target faces under the food and mosaic conditions were calculated as the score of each participant's food preference under each presentation condition. Then, the relationships between the food preference and DEBQ scores were assessed using Pearson's product–moment correlation coefficients. Furthermore, we statistically tested the differences in the associations between food preference and DEBQ score across homeostatic state conditions by performing a series of multiple regression analyses. We used the DEBQ score (restrained eating, emotional eating, or external eating; mean centered), homeostatic state, and the interaction between DEBQ score and homeostatic state as the independent variables and the food preference (under the subliminal or

supraliminal condition) as the dependent variable. The coefficients of the interactions were evaluated using *t*-tests (two-tailed). Although our hypothesis only concerned the relationship between food preference under the subliminal condition and external eating tendency, as described in the Background section, we also exported other relationships for descriptive purposes.

Results were considered statistically significant at $p < 0.05$.

References

1. Sato W, Sawada R, Kubota Y, Toichi M, Fushiki T. Unconscious affective responses to food. PLoS One. 2016;11:e01609562016.

2. van Strien T, Frijters JER, Bergers GPA, Defares PB. The Dutch Eating Behavior Questionnaire (DEBQ) for assessment of restrained, emotional, and external eating behavior. Int J Eat Disord. 1986;5:295–315.

3. Imada S. Shokukodo nikansuru shinrigakutekikenkyu (3): Nihongoban DEBQ shitsumonshino hyojunka. Hiroshimashudaironshu. 1994;34:281–91.

4. Esteves F, Öhman A. Masking the face: Recognition of emotional facial expressions as a function of the parameters of backward masking. Scand J Psychol. 1993;34:1–18.

5. Peryam DR, Pilgrim FJ. Hedonic scale method of measuring food preferences. Food Technol. 1957;11:9–14.

6. Murphy ST, Zajonc RB. Affect, cognition, and awareness: Affective priming with optimal and suboptimal stimulus exposures. J Pers Soc Psychol. 1993;64:723–39.

7. Sato W, Aoki S. Right hemispheric dominance in processing of unconscious

negative emotion. Brain Cogn. 2006;62:261–6.

8. Eriksen CW. Discrimination and learning withtout awareness: A methodological survey and evaluation. Psychol Rev. 1960;67:279–300.

9. Kirk RE. Experimental design: Procedures for the behavioral sciences. (3rd ed.) California: Brooks/Cole; 1994.
