# Supplementary material for: Homeostatic modulation on unconscious hedonic responses to food
Source: BMC Res Notes. 2017 Oct 26;10:511. doi: 10.1186/s13104-017-2835-y (PMC5658914; doi:10.1186/s13104-017-2835-y)
Supplement: Supplementary file 2 — Additional file 2. Supplementary findings. Findings from additional analysis. [file 13104_2017_2835_MOESM2_ESM.pdf]

Additional File 2: Supplementary Findings

Because the interactions between homeostatic state and stimulus type could be confounded with several factors, including sex (female/male) and age of the participant, food type (fast food/Japanese diet), and visual field (left/right), we conducted additional analyses of these factors. To simplify the analyses, differences in preference ratings between the food and mosaic conditions were used as the dependent measure. Thus, the main effects of homeostatic state (hungry/satiated) in the following analyses correspond to the interactions between homeostatic state and stimulus type in the main analyses (our effect of interest).

The mean (± standard error) preference ratings across sex, visual field, food type, homeostatic state, stimulus type, and presentation condition are shown in Figure S1.

Under the subliminal presentation condition, a four-way repeated-measures analysis of covariance (ANCOVA) was conducted with homeostatic state and sex as between-subject factors and food type and visual field as within-subject factors with age as a covariate. This analysis confirmed the significant main effect of homeostatic state ($F(1,51) = 7.76$, $p < 0.01$, $\eta_p^2 = 0.13$), indicating that the preference differences between the food and mosaic conditions differed across homeostatic conditions (higher under the hungry condition than under the satiated condition).

Because several additional interactions were found, we conducted further analyses. Because the initial analysis revealed no significant main effects or interactions related to sex or age ($F(1,51) < 0.80$, $p > 0.1$), we omitted this factor and covariate and performed a three-way ANOVA with homeostatic state as a between-subject factor and food type and visual field as within-subject factors. The analysis revealed a significant main effect of homeostatic state ($F(1,54) = 8.56$, $p <$

0.01). Additionally, we found a significant two-way interaction between homeostatic state and visual field ($F(1,54) = 4.49$, $p < 0.05$) and a significant three-way interaction ($F(1,54) = 9.88$, $p < 0.005$). We found no other significant main effects or interactions ($F(1,54) < 2.53$, $p > 0.1$).

Follow-up analyses were conducted for the three-way interaction to specify the effect of homeostatic state under different conditions. The simple-simple main effect of homeostatic state was significant for fast food stimuli presented in the right visual field ($F(1,216) = 14.73$, $p < 0.001$) and marginally significant for Japanese diet stimuli presented in the left visual field ($F(1,216) = 3.04$, $p < 0.05$), but not for fast food stimuli presented in the left visual field or Japanese diet stimuli presented in the right visual field ($F(1,216) < 2.18$, $p > 0.1$).

We performed a four-way repeated-measures ANCOVA with homeostatic state and sex as between-subject factors, food type and visual field as within-subject factors, and age as a covariate under the supraliminal presentation condition. Similar to the findings under the subliminal condition, the analysis confirmed a significant main effect of homeostatic state ($F(1,51) = 133.95$, $p < 0.001$, $\eta_p^2 = 0.22$), indicating that the preference differences between the food and mosaic conditions differed across homeostatic conditions (i.e., higher under the hungry than under the satiated condition).

Because the initial analysis revealed no significant main effects or interactions related to age or visual field ($F(1,51) < 1.97$, $p > 0.1$), we omitted those effects from further analysis and performed a three-way ANOVA with homeostatic state and sex as between-subject factors and food type as a within-subject factor. The analysis revealed a significant main effect of homeostatic state ($F(1,52) = 13.35$, $p < 0.001$).

Besides, there were a significant main effect of food type ($F(1,52) = 4.34$, $p < 0.05$) and significant interactions between homeostatic state and food type ($F(1,52) = 8.73$, $p < 0.005$) and sex and food type ($F(1,52) = 4.25$, $p < 0.05$). We found no other significant main effects or interactions ($F(1,54) < 1.78$, $p > 0.1$).

Follow-up analyses of the interaction between homeostatic state and food type were conducted to determine the effect of homeostatic state under different conditions. The simple main effects of homeostatic state were significant for both fast food and Japanese diet stimuli ($F(1,104) = 18.59$, $p < 0.001$; $F(1,104) = 7.45$, $p < 0.01$, respectively).

In summary, the modulatory effect of homeostatic state on hedonic responses to food versus mosaic stimuli under the subliminal and supraliminal conditions were confirmed when other factors, such as sex, age, food type, and visual field, were included in the analysis. In addition, we found that the effect may be modulated by some factors, including food type and visual field. However, these results are preliminary because of limitations in our experimental design. For example, we presented only four subcategories of fast food and Japanese diet, which may not adequately represent food types. Generating hypotheses regarding these factors based on our findings may provide interesting avenues for future research.

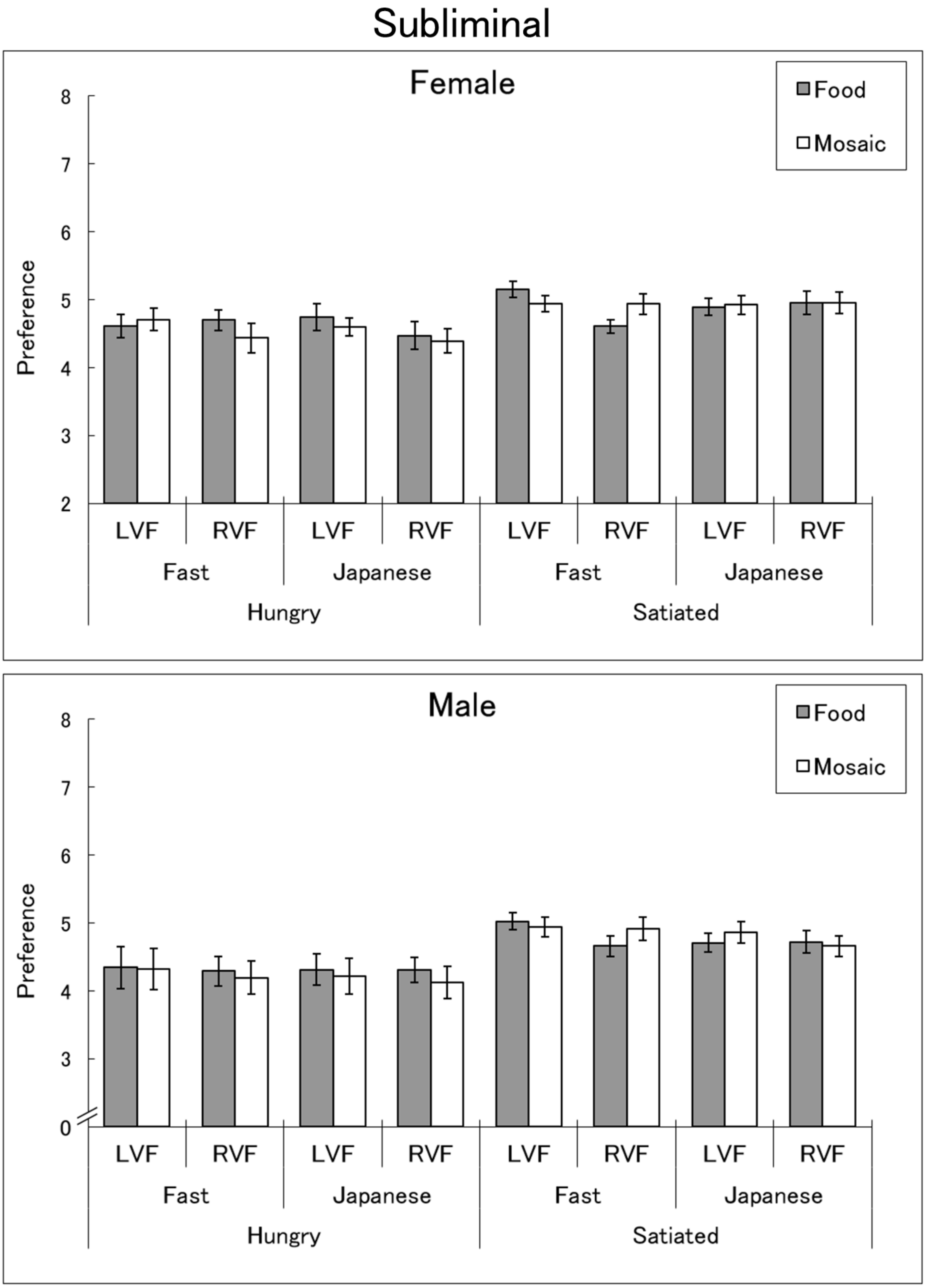

Figure S1. Continued on next page.

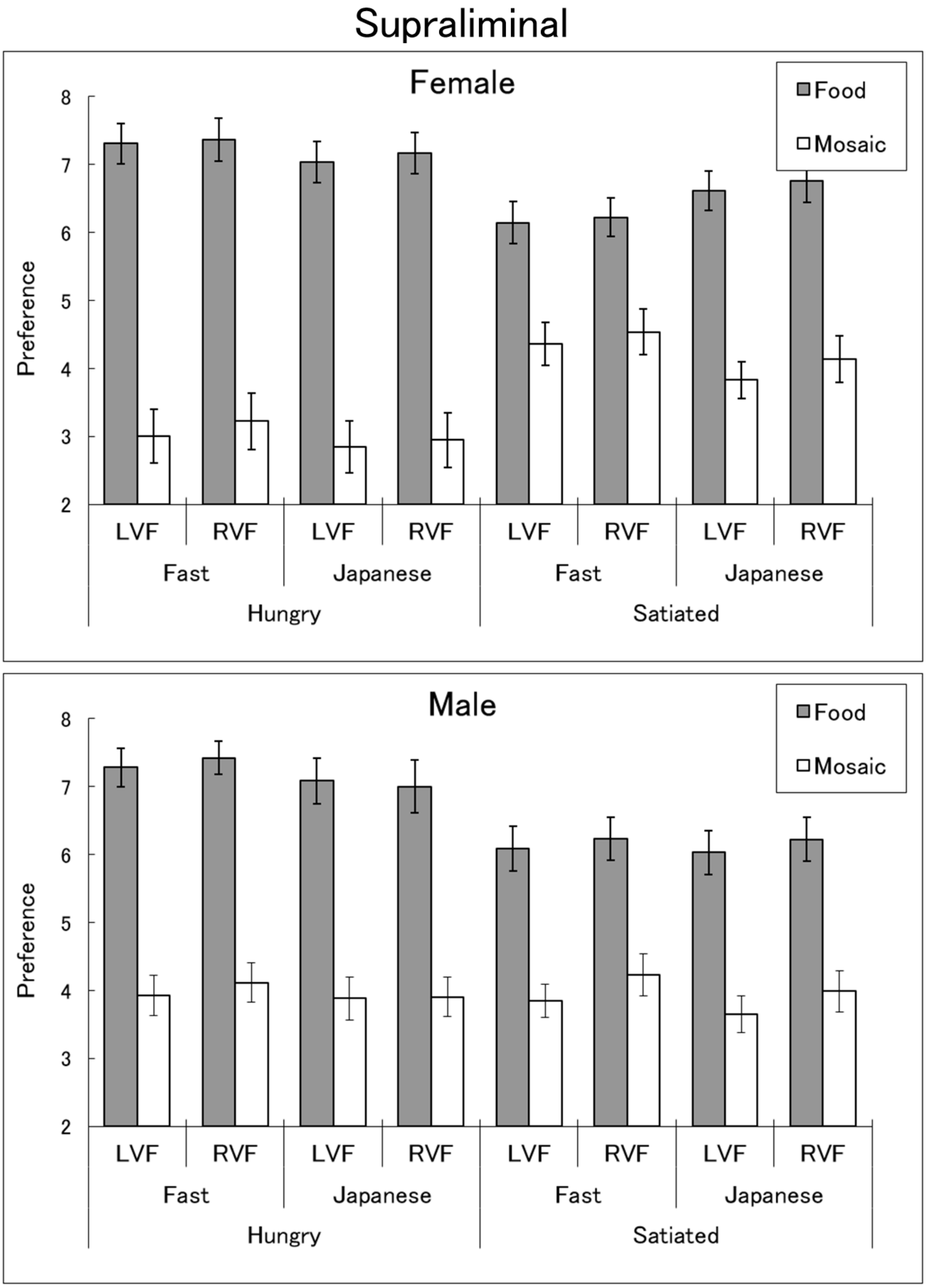

Figure S1. Mean (± standard error) preference ratings. LVF = left visual field; RVF = right visual field. Fast = Fast food; Japanese = Japanese diet.
