# Supplementary material for: Homeostatic modulation on unconscious hedonic responses to food
Source: BMC Res Notes. 2017 Oct 26;10:511. doi: 10.1186/s13104-017-2835-y (PMC5658914; doi:10.1186/s13104-017-2835-y)
Supplement: Supplementary file 3 — Additional file 3: Table S1. Mean (± standard error) scores and the correlation coefficients. [file 13104_2017_2835_MOESM3_ESM.pdf]

**Additional File 3: Supplementary Table 1.** Mean (± standard error) scores and the correlation coefficients.

| | Scale | Mean (± standard error) | | Correlation | | | | | | | |
|---|---|---|---|---|---|---|---|---|---|---|---|
| | | Hungry | Full | 1 | | 2 | | 3 | | 4 | |
| | | | | Hungry | Full | Hungry | Full | Hungry | Full | Hungry | Full |
| 1 | Subliminal preference difference | 0.1 (0.0) | -0.1 (0.0) | | | | | | | | |
| 2 | Supraliminal preference difference | 3.7 (0.3) | 2.2 (0.3) | -0.07 | 0.30 | | | | | | |
| 3 | Restrained eating | 2.5 (0.2) | 2.2 (0.1) | -0.38 * | 0.06 | 0.16 | -0.10 | | | | |
| 4 | Emotional eating | 2.7 (0.2) | 2.4 (0.1) | 0.20 | -0.09 | 0.22 | -0.12 | 0.17 | 0.18 | | |
| 5 | External eating | 3.6 (0.1) | 3.5 (0.1) | 0.35 * | -0.19 | 0.25 | 0.04 | -0.30 | -0.26 | 0.25 | 0.70 * |

* $p < 0.05$.
